# A dynamic model for health screening: misperceptions, feedback and long term trends in screening mammography

**DOI:** 10.1186/1748-5908-10-S1-A50

**Published:** 2015-08-20

**Authors:** Ozge Karanfil, John D Sterman

**Affiliations:** 1 MIT Sloan School of Management, System Dynamics Research Group, Cambridge, MA 02142, USA

## 

Implications of widespread mammography screening remain controversial, and major health organizations in the US adopt different guidelines reflecting significant variations in actual practice. Literature suggests that implementation of routine screening over the past 30 years has incurred less benefit and more harms than is formerly believed.

The classical approach to setting guidelines is based on the statistical paradigm of Type-I and Type-II errors, seeking to find an evidence-based balance between sensitivity (and thus the risk of false positives) and specificity (and the risk of false negatives), given the costs and benefits of different outcomes.

Clearly, however, a wide range of other considerations play important roles in determining both the formal guidelines and how these are implemented, interpreted and acted upon by providers, payers, patient advocacy groups, and other stakeholders and actors. Science and scientific evidence, including the processes that generate evidence, are embedded in and not independent of a social, economic and political system.

In this study we develop the first explicit and integrated, broad boundary feedback theory around the dynamics of health screening. The theory we develop includes a decision-theoretic core around the costs and benefits of screening including the fundamental tradeoff between sensitivity and specificity. It also includes some of the sociopolitical feedbacks that condition formal guidelines, public perception, and the actual practice. To provide context, we use the mammography case as the motivating example, but our model is generic enough to be applicable to other contexts of population health screening, such as the prostate-specific antigen (PSA) screening.

Our model is tightly grounded in empirical evidence base, and we use a mix of qualitative/quantitative methods and mainly system dynamics to explain the dynamic nature of the medical screening problem within the US context. This study is part of my PhD dissertation and funded by MIT.

